# Brain networks of perceptual decision-making: an fMRI ALE meta-analysis

**DOI:** 10.3389/fnhum.2014.00445

**Published:** 2014-06-19

**Authors:** Max C. Keuken, Christa Müller-Axt, Robert Langner, Simon B. Eickhoff, Birte U. Forstmann, Jane Neumann

**Affiliations:** ^1^Faculty of Social and Behavioural Science, Cognitive Science Center Amsterdam, University of AmsterdamAmsterdam, Netherlands; ^2^Max Planck Institute for Human Cognitive and Brain SciencesLeipzig, Germany; ^3^Institute of Clinical Neuroscience and Medical Psychology, Heinrich Heine University DüsseldorfDüsseldorf, Germany; ^4^Research Centre Jülich, Institute of Neuroscience and Medicine (INM-1)Jülich, Germany; ^5^Leipzig University Medical Center, IFB Adiposity DiseasesLeipzig, Germany

**Keywords:** decision-making, meta-analysis, fronto-parietal-basal ganglia

## Abstract

In the recent perceptual decision-making literature, a fronto-parietal network is typically reported to primarily represent the neural substrate of human perceptual decision-making. However, the view that only cortical areas are involved in perceptual decision-making has been challenged by several neurocomputational models which all argue that the basal ganglia play an essential role in perceptual decisions. To consolidate these different views, we conducted an Activation Likelihood Estimation (ALE) meta-analysis on the existing neuroimaging literature. The results argue in favor of the involvement of a frontal-parietal network in general perceptual decision-making that is possibly complemented by the basal ganglia, and modulated in substantial parts by task difficulty. In contrast, expectation of reward, an important aspect of many decision-making processes, shows almost no overlap with the general perceptual decision-making network.

## Introduction

Many of our decisions in everyday life rely on our senses and how quickly and accurately we extract information from our environment. Consider, for example, driving down the highway on a motorcycle while it starts to rain. Soon, your visibility is significantly reduced due to accumulating raindrops on your helmet visor and it becomes harder to see if the car in front of you is slowing down and whether you need to slow down as well.

Which brain areas are involved in these kinds of perceptual decision-making processes is a key question in cognitive neuroscience (Schall, 2001; Krawczyk, 2002; Platt, 2002; Romo and Salinas, 2003; Gold and Shadlen, 2007; Heekeren et al., 2008; Ding and Gold, 2013). While most of the current insights stem from single-unit recordings in monkeys, an increasing number of functional magnetic resonance imaging (fMRI) studies have addressed the neural correlates of perceptual decision-making in humans (Kim and Shadlen, 1999; Schall, 2001; Shadlen and Newsome, 2001; Krawczyk, 2002; Platt, 2002; Glimcher, 2003; Romo and Salinas, 2003; Romo et al., 2003; Gold and Shadlen, 2007; Churchland et al., 2008; Heekeren et al., 2008). These studies frequently employ simple perceptual discrimination tasks, which typically feature two or more forced-choice alternatives at varying levels of difficulty (Gold and Shadlen, 2007). A straightforward way of manipulating the difficulty is to change the amount of sensory evidence provided by the experimental stimuli. For example, the number of coherently moving dots in the often-used “*random dot motion paradigm*” may be reduced in order to make the judgment on the direction of motion considerably more difficult (Britten et al., 1992; Palmer et al., 2005).

Evidently, a key question in this line of research is which brain areas or networks are involved in choices that are based on varying degrees of sensory evidence. Neurophysiological evidence in monkeys suggests that the decision-forming process for such simple perceptual decision-making tasks starts off with the integration of sensory evidence for each choice by lower-level sensory neurons (Heekeren et al., 2008). The decision is then thought to be computed in higher-order cortical regions by comparing the difference in amount of sensory information for each choice. Once enough evidence in favor of a certain choice has been accumulated, the information is passed on to the motor system, thereby enabling the execution of an action associated with that specific decision (Gold and Shadlen, 2001; Heekeren et al., 2008). Previous studies have argued for a fronto-parietal network to subserve this functionality and hence to enable simple perceptual decision-making (Ho et al., 2009; Kable and Glimcher, 2009; Li et al., 2009; Mulder et al., 2012).

The presumption that perceptual decision-making is primarily implemented by a fronto-parietal network, however, has been challenged by recent neuro-computational models. These models state that the basal ganglia (BG) are likewise essential for the computation of perceptual decisions and should not be neglected in theorizing (Bogacz, 2007; Ding and Gold, 2013). The BG is a collection of subcortical nuclei that anatomically consist of the striatum and pallidum. Additionally, the subthalamic nucleus and the substantia nigra are functionally considered to be part of the BG (Federative Committee on Anatomical Terminology, 1998). It has been argued that the BG as a whole implement a central gating mechanism by evaluating the evidence of each choice alternative facilitating the appropriate behavioral response for the alternative with the most supporting evidence (Lo and Wang, 2006; Bogacz and Gurney, 2007; Frank et al., 2007). More specifically, the model by Bogacz and Gurney (2007) proposes that the striatum is involved in encoding certain actions whereas the subthalamic nucleus inhibits the output to the thalamus until enough information is accumulated. In line with this reasoning, several fMRI studies have shown an involvement of the striatum in flexibly adapting the response regime in such simple perceptual decision-making tasks (Forstmann et al., 2008a, 2010a,b; van Maanen et al., 2011). Other studies argue for the involvement of the subthalamic nucleus in task-switching or in mediating the decision-threshold under stimulus conflict (Cavanagh et al., 2011; Mansfield et al., 2011). In addition, there is a large body of literature on the involvement of the BG in reward-based decision-making (Kawagoe et al., 1998; Tanaka et al., 2004; Liu et al., 2011; Mulder et al., 2013). While these results point toward an involvement of subcortical brain structures in several aspects of perceptual decision-making, it remains unclear if the BG are also involved in decision-making aspects such as task difficulty.

Finally, several recent reviews hypothesize that the perceptual decision-making network serves as a core network that can be recruited for other forms of decision-making such as reward-based decision-making (Gold and Shadlen, 2007; Heekeren et al., 2008). However, whether or not this theory holds, has yet to be shown. By combining the literatures on perceptual and reward-based decision-making, it becomes possible to test whether reward-based decision-making recruits a similar network as does perceptual decision-making.

The present study set out to address the following questions:

1. Which cortical and subcortical brain areas are consistently involved in simple perceptual decision-making?
2. To what extent is this perceptual decision-making network modulated by task difficulty?
3. To what extent does the task-general network for simple perceptual decision-making overlap with a reward-based decision-making network?

In order to answer these questions a number of Activation Likelihood Estimation (ALE) meta-analyses of fMRI studies were conducted on simple perceptual decision-making tasks as well as reward-based decision-making. Such meta-analyses go beyond qualitatively pooling results from diverse neuroimaging experiments by quantitatively modeling reported brain coordinates and statistically testing their convergence across studies in standard brain space (Turkeltaub et al., 2002; Neumann et al., 2008; Eickhoff et al., 2009, 2012).

## Methods

A comprehensive search for relevant neuroimaging studies in the field of perceptual decision-making was carried out using the PubMed database (www.pubmed.org). The three main keywords utilized were “fMRI,” “neural,” and “brain.” Each of these keywords was entered in combination with general keywords (e.g., “perceptual decision-making”) as well as more specific keywords (e.g., “random dot motion” see Table 1 for all keywords). Based on the information contained in the abstracts of all papers returned, empirical studies were selected to meet the following inclusion criteria: (1) Studies were published in peer-review English language journals between January 2000 and March 2012; (2) they employed fMRI in healthy adults; (3) participants engaged in simple decision-making tasks with at least two alternatives that did not explicitly require higher-order cognitive functions such as language or memory; (4) studies reported a Task > Control contrast or a Hard > Easy contrast of the experimental task; and (5) they reported whole-brain activations as 3D coordinates in stereotactic space of Talairach or the Montreal Neurological Institute (MNI). Subsequently, the full texts of all applicable studies were read to confirm the valid inclusion in the meta-analysis. Finally, the included empirical studies were cross-referenced, and the whole selection process was repeated for the newly obtained empirical papers.

**Table 1:** **All the keyword combinations used to search the PubMed database where the first column was combined with the second column**.

fMRI	Random dot motion	(50)
	Perceptual decision-making	(122)
	Faces and houses	(79)
	Speed accuracy tradeoff	(10)
	Speed Accuracy	(296)
	Speed-accuracy-tradeoff	(8)
	Speed-accuracy	(21)
	Perceptual discrimination	(326)
	Perceptual judgment	(85)
	Random dot kinematogram	(2)
	Motion discrimination	(165)
	Evidence accumulation	(219)
	Perception noise	(421)
Neural	Random dot motion	(117)
	Perceptual decision-making	(234)
	Speed accuracy tradeoff	(21)
	Evidence accumulation	(356)
Brain	Random dot motion	(267)
	Perceptual decision-making	(402)
	Speed accuracy tradeoff	(29)

The number in the parentheses indicates the total number of papers returned for the corresponding keyword combination. The other combinations did not yield new results.

Additionally, to increase the number of possible relevant empirical studies, all abstracts returned by PubMed were scanned for reviews. This was done by searching the initial list of abstracts returned by PubMed for the keywords “review,” “summary,” and “summarize.” Based on the abstracts of the obtained reviews, only reviews that covered the topic of decision-making were then cross-referenced, and the whole selection process was repeated for the newly obtained empirical papers.

**Figure 1:**
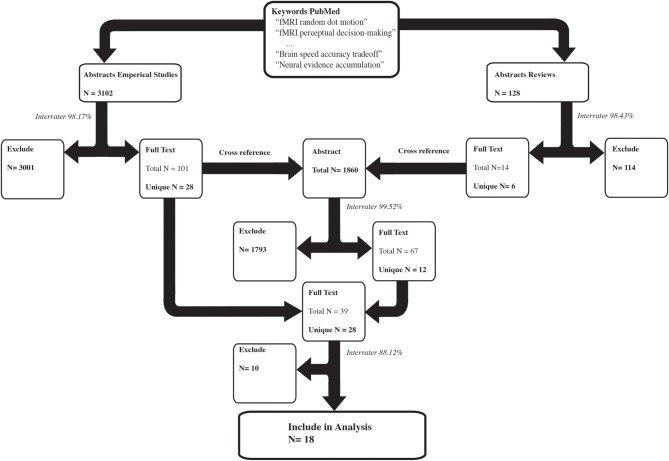
**The selection procedure for the inclusion of empirical studies**. The left arm shows the selection process of the empirical studies based on the abstracts. The right arm shows the selection process of the review papers based on the abstracts. The number of results per selection stage is reported in bold. Several keywords resulted in the inclusion of the same study, which is reflected by the total N. Subsequently only the unique papers were used in the next selection step. The interrater congruency between the two independent raters is reported in italics. For instance, of the 3102 empirical abstracts, both raters independently agreed on 98.17% of the abstracts to either exclude them or to read the full text. The remaining abstracts were discussed and a consensus was reached on whether to exclude the abstract or to read the full text.

Two independent raters completed the entire article inclusion procedure, and only articles that both raters agreed on were included in the final sample. See Figure 1 for an overview of the selection and inclusion process and Table 2 for the included studies. Note that several studies reported both Task > Control and Hard > Easy contrasts.

**Table 2:** **A summary of the included studies per Activation Likelihood Estimate (ALE) analyses**.

**Contrast**	**Authors**	**Task**	**Number of subjects**	**Number of Foci**	**Statistical threshold**	**Smoothing FWHM (mm)**
Task > Control	Banko et al., 2011	Face recognition	16	4	FDR 0.05	8
	Bode et al., 2012	Object recognition	14	1	FWE 0.001	8
	Ivanoff et al., 2008	RDM motion discrimination	22	49	FDR 0.05	4
	Kahnt et al., 2011	Gabor patch orientation discrimination	20	7	Unc. 0.0001	6
	Lewis et al., 2000	Auditory motion discrimination	10	9	Unc. 1 × 10^−5^	4
		RDM discrimination	9	10	Unc. 1 × 10^−11^	4
	Lundblad et al., 2011	Tactile motion discrimination	16	24	FWE 0.05	8
	Singh and Fawcett, 2008^*^	RDM discrimination	7	30	Cluster 0.01	5
	Snyder et al., 2011	RDM discrimination	10	15	FDR 0.05	8
		Object recognition	10	11	FDR 0.05	8
Hard > Easy	Banko et al., 2011	Face recognition	16	10	FDR 0.05	8
	Bode et al., 2012	Object recognition	14	1	FWE 0.001	8
	Fleming et al., 2010^*^	Face vs house discrimination	14	2	Cluster 0.001	8
	Heekeren et al., 2004	Face vs house discrimination	12	14	Unc. 0.0001	8
	Heekeren et al., 2006^*^	RDM discrimination	8	5	Unc. 0.005	8
	Ho et al., 2009	RDM discrimination	11	11	FDR 0.05	4
	Kayser et al., 2010a	RDM discrimination	5	22	Unc. 0.0001	5
		Color discrimination	5	18	Unc. 0.0001	5
	Kayser et al., 2010b	RDM discrimination	6	25	Unc. 0.0001	6
	Noppeney et al., 2010	Object recognition	19	9	Cluster 0.05	8
	Philiastides and Sajda, 2007^*^	Face vs. cars discrimination	12	5	Cluster 0.05	8
	Sunaert et al., 2000	RDM discrimination	8	5	Cluster 0.05	10
	Tosoni et al., 2008^*^	Face vs. house discrimination	12	18	Unc. 0.05	n.s.

Several studies conducted multiple experiments and each experiment is reported as a separate contrast. FWHM, full width at half maximum; RDM, random dot motion; Unc, uncorrected; FWE, familywise error rate; FDR, false discovery rate; n.s., not stated.

* Coordinates acquired via personal communication.

For answering the third question of this paper (i.e., the specificity of the task-general network for simple perceptual decision-making tasks), a separate meta-analysis was conducted on reward-based decision-making to assess the overlap as well as the difference between perceptual and reward-based decision-making. Selection of relevant studies was based on a recently published ALE meta-analysis on reward processing in the brain conducted by Liu et al. (2011). For this meta-analysis, the authors identified 142 neuroimaging studies that examined brain activation in reward-related decision-making tasks in healthy adults. In order to ensure direct comparability with the perceptual decision-making meta-analysis, only a subset of these studies was chosen here. Specifically, only studies reporting Reward > Control contrasts were considered. In these studies, the reward condition contained a cue informing the participant that one of the choice alternatives would result in a larger reward, whereas in the control condition the participant received a neutral cue. In addition, only studies that assessed the BOLD response in a time window comparable to the perceptual decision-making tasks (i.e., the actual decision process rather than later components such as the period between making a response and receiving feedback) were selected.

See Table 3 for the studies included in the meta-analysis on reward-based decision-making.

**Table 3:** **The studies selected from the meta-analysis of Liu et al. (2011)**.

**Contrast**	**Authors**	**Task**	**Number of subjects**	**Number of foci**
Reward anticipation > Control	Adcock et al., 2006	Monetary incentive encoding	12	17
			12	17
	Knutson et al., 2001	Monetary incentive delay	8	2
	Knutson et al., 2003	Monetary incentive delay	12	7
	Bjork, 2004	Monetary incentive delay	12	13
	Fukui et al., 2005	Iowa gambling task	14	1
			14	1
	Juckel et al., 2006	Monetary incentive delay	10	9
			10	18
	Satterthwaite et al., 2007	Gambling	26	6
	Ströhle et al., 2008	Monetary incentive delay	10	7
	Xue et al., 2009	Cubs task	13	9
	Wrase et al., 2007	Monetary incentive delay	16	3
			16	2

Several studies conducted multiple experiments and each experiment is reported as a separate contrast.

### Activation likelihood estimation

ALE analyses were performed using the BrainMap application GingerALE, version 2.3 (http://brainmap.org/ale/). All activation foci of the included studies that were originally reported in Talairach space were converted to the MNI stereotactic space using the Lancaster et al. (2007) transformation algorithm. Within ALE, these activation foci are modeled as the center of a three-dimensional Gaussian probability distribution reflecting the spatial uncertainly associated with the respective neuroimaging findings. Combining these distributions within and across experiments, a statistical whole-brain map is created that yields an estimate of the activation likelihood (i.e., ALE value) for each voxel, based on all reported activation foci (Eickhoff et al., 2009). In order to confine the number of inflated ALE values arising from experiments reporting many proximate activation foci, a non-additive ALE method was chosen (Turkeltaub et al., 2012). Utilizing this approach, significant ALE values are less likely to be caused by simple within-experiment effects, but reflect the actual concordance in activation patterns between the different experiments. Furthermore, the FWHM of the 3D probability distribution was estimated per individual study, resulting in a higher specificity of the actual overlap between studies (Eickhoff et al., 2009).

Subsequently, to test against the null hypothesis of spatial independence of activation foci, the analytical approach based on a non-linear histogram integration as described by Eickhoff et al. (2012) was employed. To correct for multiple comparisons, a cluster-level approach with a cluster-forming threshold of *p* = 0.05 was used. To estimate a null-distribution of cluster sizes, a random set of experiments was created with the same characteristic as the actual data but with random coordinates. For this random set, the same ALE analysis was performed and this process was repeated 10,000 times to create a null-distribution of cluster sizes. In the following, all clusters that exceeded the critical threshold to control for the cluster-level family wise error rate at *p* < 0.05 are reported.

### Testing for overlap between different task aspects

The overlap between the Task > Control contrast, the Hard > Easy contrast, and the Reward > Control contrast was analyzed by computing the pairwise minimum conjunction of the respective ALE maps (Nichols et al., 2005), whereas unique clusters for each contrast were identified by pairwise subtraction analyses (Eickhoff et al., 2011). The subtraction analysis entailed that all experiments that contributed to the initial contrast were pooled and randomly divided into two equally sized groups. The ALE values for these two randomly divided groups were then calculated, and the difference between these ALE values was recorded per voxel. This process was repeated 10,000 times and resulted in a null-distribution for the difference in ALE values. The actual observed difference between the two contrasts was then compared to the null-distribution and resulted in a *p*-value map. This map was statistically thresholded at a level of *p* < 0.05, resulting in a set of areas that were reliably associated with one of the two networks but not the other.

Anatomical labels for the final activation cluster locations were determined using the Anatomy Toolbox and the Harvard-Oxford atlas as implemented in FSL, version 5.0.2 (Eickhoff et al., 2005, 2006, 2007; Choi et al., 2006; Desikan et al., 2006; Makris et al., 2006; Caspers et al., 2008).

## Results

**Figure 2:**
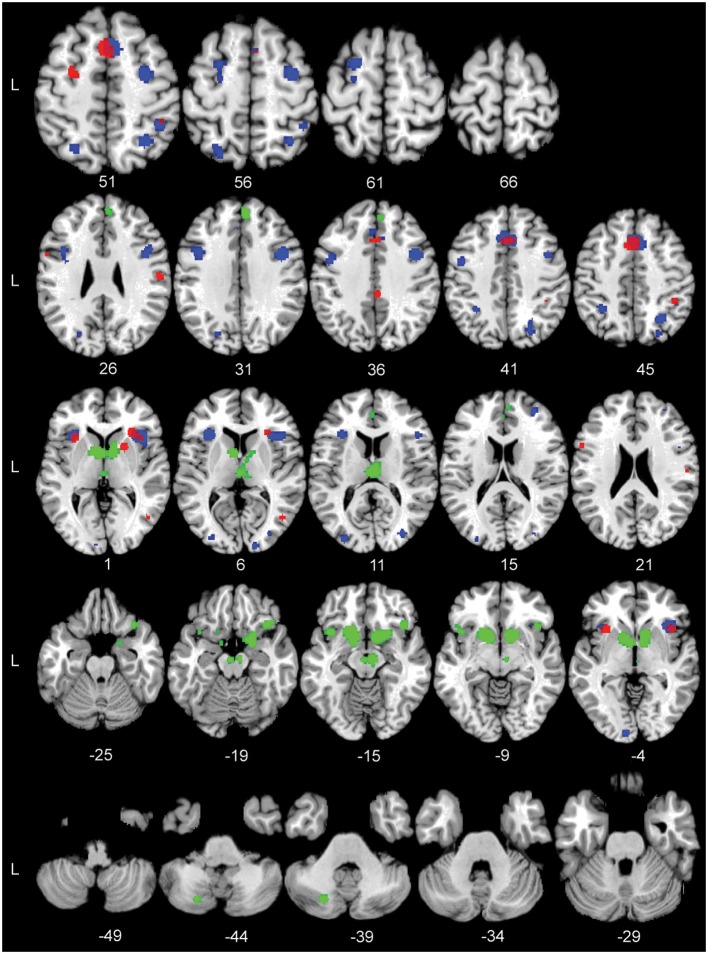
**The significant Activation Likelihood Estimate (ALE) clusters for the three separate ALE analyses in standard Montreal Neurological Institute space**. Red: Task > Control; blue: Hard > Easy; green: Reward > Control. Numbers indicate Z coordinates in MNI space.

Figure 2 shows the location of the activation clusters revealed by the three individual meta-analyses. The ALE meta-analysis for Task > Control, based on 10 contrasts and 160 foci, revealed 12 significant clusters. The largest clusters were located in the bilateral pre-supplementary motor area (pre-SMA), bilateral anterior insula, the right putamen, the right opercular supramarginal area (PFop, Triarhou, 2007; Caspers et al., 2008) located in the supramarginal gyrus, and the left middle frontal gyrus (MFG). See Table 4 for the coordinates of all the 12 clusters that form this perceptual decision-making network.

**Table 4:** **Significant activation clusters of the Activation Likelihood Estimate (ALE) analyses**.

**Contrast**	**Region**	**Volume(mm^3^)**	***x***	***y***	***z***	**ALE(x10^3^)**	**Number of studies/Foci per cluster**
Task > Control (minimum cluster size 304mm^3^)	L pre-SMA	2560	−2	18	46	19.3	4/10
	R pre-SMA		6	12	58	8.8	
	R insula; anterior part	1304	30	24	2	17.05	4/5
	R insula; anterior part		40	18	−4	14.4	
	L insula; anterior part	768	−32	18	−2	14.7	3/3
	R putamen	448	22	8	2	15.2	2/2
	R inferior parietal lobule (PFop)	432	56	−16	24	14.7	2/2
	L middle frontal gyrus	432	−28	−4	52	11.2	1/1
	R posterior cingulate gyrus	312	4	−36	34	11.6	2/2
	R inferior parietal lobule (hIP2)	312	42	−42	46	10.7	2/2
	R anterior occipital sulcus (hOC5)	304	46	−66	4	12.1	2/2
	L inferior frontal gyrus; p. opercularis	304	−56	10	22	10.4	2/2
Hard > Easy (minimum cluster size 320mm^3^)	R pre-SMA	4040	2	18	46	24.7	9/10
	R insula; anterior part	3560	38	20	0	20.1	9/9
	R inferior frontal gyrus; p. triangularis		50	22	12	9.7	
	R pre-central gyrus	2328	42	4	32	25.4	6/6
	R angular gyrus; hIP3	2168	28	−60	46	14	6/7
	R superior occipital gyrus; SPL		24	−72	42	10.5	
	L inferior frontal gyrus; p. opercularis	1904	−40	8	28	11.4	5/6
	L pre-central gyrus		−46	−2	38	11	
	L pre-central gyrus		−38	−2	26	7.9	
	L insula; anterior part	1840	−32	22	4	17.5	6/6
	R pre-central gyrus	1448	32	−6	54	15.3	4/5
	L superior frontal gyrus	1008	−22	4	60	11.6	3/4
	L superior frontal gyrus		−24	−10	58	9	
	L superior parietal lobule (SPL)	760	−26	−66	54	10.5	2/3
	R inferior parietal lobule (hIP3)	680	42	−46	52	12.3	2/3
	L inferior parietal lobule (hIP3)	552	−30	−50	44	12.4	2/2
	L middle occipital gyrus (hOC3v)	520	−30	−90	10	11.4	1/1
	R middle occipital gyrus	440	32	−84	10	11.3	2/2
	L calcarine gyrus	432	−10	−96	−4	12	2/2
	R calcarine gyrus	360	16	−98	4	11.5	2/2
	R middle frontal gyrus	360	32	46	16	10.6	2/2
	L superior occipital gyrus	320	−22	−76	30	9	2/2
Reward Anticipation > Control (minimum cluster size 288mm^3^)	R caudate nucleus	15208	12	10	−10	33	8/48
	L putamen		−12	8	−10	26.5	
	L caudate nucleus		−6	2	0	23	
	R pallidum		10	4	−2	17.7	
	R rectal gyrus		22	12	−16	17.2	
	L thalamus		0	−18	10	16	
	R amygdala		22	2	−20	15	
	L amygdala		−14	2	−16	14.3	
	R substantia nigra	1664	8	−16	−16	19.6	2/5
	L mammillary body		−2	−16	−18	14.1	
	R inferior frontal gyrus; p. orbitalis	1568	36	22	−22	14.4	4/5
	R inferior frontal gyrus; p. orbitalis		42	22	−14	13	
	R superior medial gyrus	1040	6	46	30	15.2	3/4
	L insula; anterior part	1000	−38	14	−16	14.5	2/4
	L cerebellum; lobule VII crus II	448	−22	−74	−42	13.8	1/2
	L anterior cingulate gyrus	440	0	42	12	10	2/3
	R superior medial frontal gyrus		6	52	16	9.3	

Several clusters had multiple peak ALE values and are reported in ALE as subclusters. Note that these do not have a volume estimate as they are part of the main cluster.

The ALE analysis for Hard > Easy was based on 13 contrasts and 145 foci and revealed 17 separate clusters of activation. The largest clusters where found in the right pre-SMA, bilateral anterior insula, bilateral pre-central gyrus, bilateral inferior frontal gyrus (IFG), and the left superior frontal gyrus (SFG). See Table 4 for the coordinates of the 17 clusters that are part of the task-difficulty-related network.

The ALE analysis for the Reward > Control contrast, based on 14 contrasts and 112 foci, showed a network that was distributed over frontal and subcortical areas. The largest clusters were located in the bilateral striatum, right substantia nigra (SN), right IFG, left insula, and right superior medial gyrus (SMG). Notably, the Reward > Control ALE analysis did not show any parietal activation. See Table 4 for the coordinates of the local maximum ALE values.

**Figure 3:**
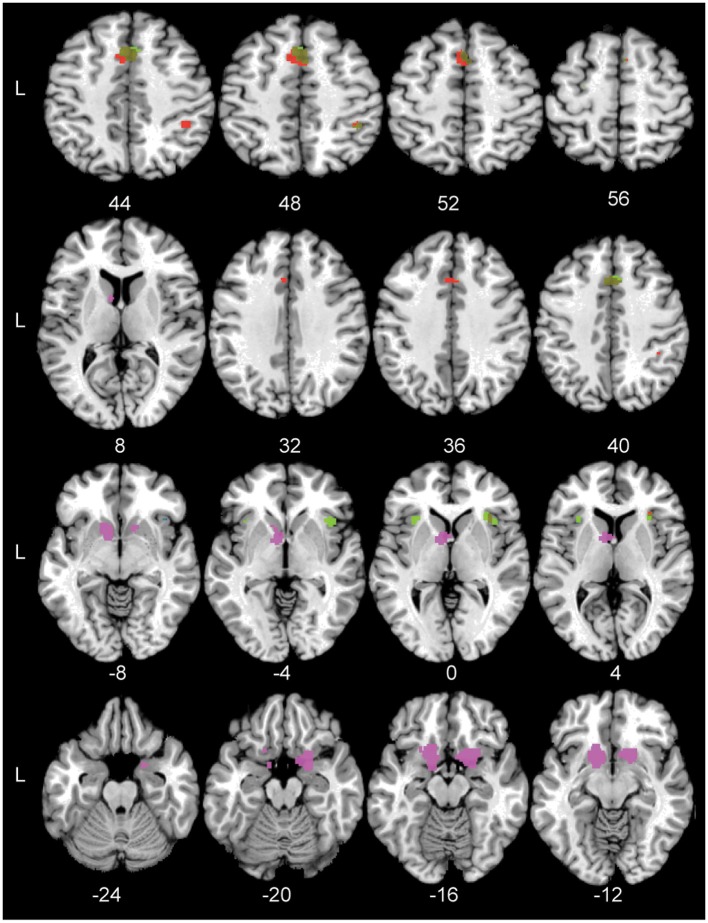
**The significant conjunction and subtraction clusters in standard Montreal Neurological Institute space**. Green: The significant conjunction clusters for the task-general network and the task difficulty network are located in the right pre-SMA, left pre-motor cortex, bilateral anterior insula, and right PFm. Blue: The significant conjunction cluster for the task-general and reward networks is located in right anterior insula. Red: The unique areas for the task-general network compared to the reward-based network are located in left pre-SMA cortex, right hIP2, and right anterior insula. Violet: The unique areas for the reward-based network are located in left nucleus accumbens and right frontal orbital cortex. Numbers indicate Z coordinates in MNI space.

Subsequently, a conjunction analysis across the Hard > Easy and Task > Control contrasts was performed in order to assess to what extent the perceptual decision-making network was modulated by task difficulty. Results of this analysis revealed a substantial overlap in the conjunction map including a cluster in the right anterior insula, right pre-SMA, left pre-motor cortex and right magnocellular supramarginal area (PFm, Triarhou, 2007; Caspers et al., 2008) in the inferior parietal lobule. Thus, considerable parts of the perceptual decision-making network were modulated by task difficulty. See Figure 3 for the location of the significant conjunction clusters. Finally, the subtraction analysis did not show any significant differences in the likelihood of activation for the Hard > Easy contrast compared to the Task > Control contrast or vice versa.

To test the specificity of the task-general network for simple perceptual decision-making, it was compared to the reward-based decision-making network. The conjunction analysis across the Task > Control and Reward > Control contrasts showed one small area of overlap in the right anterior insula. See Figure 3 for the significant conjunction cluster. The subtraction analysis revealed that the perceptual decision-making network had unique activation in the left pre-SMA, the right second human intraparietal sulcus (hIP2, Choi et al., 2006), and again in the right anterior insula, the latter cluster being located more inferior than the anterior insula cluster resulting from the conjunction. Conversely, the reward-based decision-making network showed unique involvement of the left nucleus accumbens and right orbito-frontal cortex (see Figure 3). See Table 5 for the coordinates of the local maximum ALE values revealed by the conjunction and differences between the two networks.

**Table 5:** **Significant activation clusters of the conjunction and subtraction analyses**.

**Contrast**	**Region**	**Volume (mm^3^)**	***x***	***y***	***z***	**ALE(×10^3^)**	**Number of studies/Foci per cluster**
(Task > Control) ∩ (Hard > Easy)	R pre-SMA	1712	1	17	45	18.4	9/9
	R insula; anterior part	824	40	18	−4	14.4	8/8
	R insula; anterior part		32	22	2	13.8	
	L insula; anterior part	344	−32	20	1	12.5	2/2
	R inferior parietal lobule (PFm)	104	43	−44	48	10	2/2
	L pre-motor cortex	8	−26	−10	56	7.8	
(Task > Control) ∩ (Reward > Control)	R insula; Anterior part	16	42	20	−8	8.4	
(Task > Control) > (Reward > Control)	L pre-SMA	2280	−1	15	46	3	5/8
	R superior frontal gyrus		4	18	34	2.85	
	R inferior parietal lobule (hIP2)	312	42	−42	45	2.6	2/2
	R insula; anterior part	216	32	23	6	2.91	
	R insula; anterior part		34	23	2	2.81	
(Reward > Control) > (Task > Control)	L accumbens	4456	−6	7	−9	3.72	7/14
	L amygdala		−11	−1	−16	3.54	
	L amygdala		−16	0	−15	3.19	
	L caudate nucleus		−8	5	3	3.04	
	L accumbens		−11	9	−10	2.93	
	L frontal orbital cortex		−21	12	−17	2.77	
	L putamen		−13	7	−3	2.72	
	L frontal orbital cortex		−18	20	−16	2.71	
	R frontal orbital cortex	2984	26	10	−18	3.09	8/12
	R frontal orbital cortex		26	14	−18	3.06	
	R putamen		12	11	−12	2.99	
	R frontal orbital cortex		15	12	−18	2.97	
	R parahippocampal gyrus		16	3	−17	2.83	
	R Hippocampus entorhinal cortex		19	2	−21	2.82	

Several clusters had multiple peak Activation Likelihood Estimate (ALE) values and are reported in ALE as subclusters. Note that these do not have a volume estimate as they are part of the main cluster.

## Discussion

This study set out to identify the consistent brain network underlying perceptual decision-making tasks. Such network can be obtained from quantitative meta-analyses techniques for functional imaging studies such as ALE, multi-kernel density analysis (MKDA), model-based clustering, or similar approaches that received considerable attention in the neuroimaging community in recent years (e.g., Turkeltaub et al., 2002; Neumann et al., 2005, 2008, 2011; Wager et al., 2009; Yarkoni et al., 2011; Eickhoff et al., 2012). We thus conducted an ALE meta-analysis of fMRI findings in simple perceptual decision-making experiments. In addition to identifying the task-general network for perceptual decision-making, we assessed its possible modulation by task difficulty. Finally, we tested the specificity of the perceptual decision-making network by comparing the results to a meta-analysis of reward-based decision-making experiments.

### Perceptual decision-making network

In accordance with several reviews (Schall, 2001; Gold and Shadlen, 2007; Heekeren et al., 2008), the task general network for perceptual decision-making revealed by our analysis comprised several distinct cortical areas. Frontal areas included the pre-SMA, involved in setting response thresholds (Forstmann et al., 2008b; Mansfield et al., 2011; van Maanen et al., 2011); and the left IFG pars opercularis. The latter is an area that is conventionally thought to be involved in linguistic processes, but there are several reports on its involvement in motor planning and response inhibition that could explain why a cluster of activation was found in this region (Heiser et al., 2003; Johnson and Grafton, 2003; Gough et al., 2005; Pobric, 2006; Swick et al., 2008). Finally, a recent meta-analysis further revealed that the posterior part of Brodmann area 44, which would overlap with the IFG pars opercularis, is involved in action processes (Clos et al., 2013).

In addition to these frontal areas, the bilateral anterior insula was found to be involved in the perceptual decision-making network. This area is believed to play an integrative role in perception-action coupling and is shown to be consistently involved in a wide range of paradigms (Kurth et al., 2010; Sterzer and Kleinschmidt, 2010; Chang et al., 2013; Langner and Eickhoff, 2013).

The parietal areas of the perceptual decision-making network included the PFop; an area implicated in processing spatial orientation (Mochizuki et al., 2002) and the hIP2; an area involved in processing spatial attention and numerical cognition (Wu et al., 2009; Uddin et al., 2010). Further smaller clusters were found in the posterior cingulate cortex (PCC), an area thought to be involved in task engagement or decision salience (Heilbronner et al., 2011). Additionally, a small cluster was found in the fifth human occipital area (hOC5), an area reported to be involved in the coding for visual form, motion, and the representation of objects (Vaina et al., 2001; Malikovic et al., 2006).

Importantly, as predicted by several neuro-computational models, this cortical network was complemented by subcortical activation, specifically, in the right putamen. The putamen together with the caudate forms the striatum and functions as a major input structure for the BG, as it receives a wide range of cortical inputs and is thought to be essential for action selection, learning, and reward prediction (Bogacz, 2007; Bogacz and Gurney, 2007; Chakravarthy et al., 2010; Ding and Gold, 2013). According to the model by Bogacz and Gurney (2007), the activity in the striatum reflects the encoding of certain actions. While the neuro-computational models of Bogacz and Gurney do not make explicit differential predictions for the two striatal subparts, the finding of convergence in the putamen was not surprising. Previous work has shown that the putamen is more involved in limb movements whereas the caudate might be more involved in oculomotor responses (Alexander and Crutcher, 1990; Ding and Gold, 2013). Furthermore, the putamen is known to be connected to several of the aforementioned cortical areas (Leh et al., 2007; Helmich et al., 2010).

However, it should be noted that the two studies contributing to the putamen cluster both used a passive control task where no response was necessary. In both studies subjects had to either respond with both hands or only with the left hand (Ivanoff et al., 2008; Lundblad et al., 2011). Therefore, we cannot rule out the possibility that the putamen cluster solely reflects a difference in motor-related task demands. However, based on evidence complementing our analysis, we would propose that the observed putamen likely implements more than just the motor response. Previous model-based fMRI studies have attributed activation in the putamen not solely to the motor implementation of the decision, but also to the processing of prior information regarding the stimuli (Forstmann et al., 2010b; Nagano-Saito et al., 2012). Using linear accumulation models to analyse the functional data, both studies were able to separate the actual motor response from the decision-making process and found evidence for the putamen being involved in encoding response bias (Ding and Gold, 2013). However, in our results this interpretation of the putamen cluster should be taken with caution, as the contributing coordinates for the putamen were derived without using such mathematical models and warrant further investigation.

While the involvement of the BG cannot be resolved conclusively based on the data currently available for meta-analyses, results regarding our first research question speak in favor of a perceptual decision-making network that comprises of both cortical and possibly subcortical regions.

Moreover, the analysis revealed that this network consisted of a set of nodes involved in task engagement, information encoding, response caution setting, and finally action implementation, resembling most stages necessary for making a decision (Shadlen and Newsome, 2001; Platt, 2002; Glimcher, 2003; Gold and Shadlen, 2007).

### Task difficulty effects on the perceptual decision-making network

The individual meta-analysis on task difficulty revealed that the right pre-SMA, involved in setting response thresholds (Forstmann et al., 2008b; van Maanen et al., 2011) and the left SFG an area that is reported to be involved in selective attention (Cutini et al., 2008), were part of the task difficulty network. In the parietal lobule, a significant cluster was found in the bilateral area hIP3 an area that is the possible human homolog of the macaque ventral portion of the lateral intraparietal cortex (LIP, Gillebert et al., 2013). LIP activity has been repeatedly shown to reflect the amount of information accumulated for each choice alternative (e.g., Shadlen and Newsome, 2001; Churchland et al., 2008). Based on these findings one would predict a lower BOLD response in hIP3 for hard trials compared to easy trials as the amount of available information is lower. An explanation for why the current meta-analysis found an overall higher BOLD response in hIP3 could be an increased top-down modulation of attention (Bisley, 2003; Heekeren et al., 2004; Hebart et al., 2012). Finally, bilateral early visual cortices were found to be involved in task difficulty and this activation might again be due to increased top-down modulation of attention (Spitzer et al., 1988; Sunaert et al., 2000).

The conjunction analysis showed that task difficulty modulated a subset of the perceptual decision-making network including right pre-SMA, right anterior insula, left pre-motor cortex, and right PFm. If task difficulty increases, it is expected that more attentional resources are necessary so that task performance does not suffer (Posner, 1980). The PFm might facilitate this attentional modulation as it is thought to be essential in the orientation of attention (Wu et al., 2009; Jakobs et al., 2012). With respect to our second research question, the conjunction analysis revealed that several cortical areas involved in perceptual decision making are effected by task difficulty.

### Differences between perceptual and reward-based decision-making

The final question of the present study was to test the specificity of the perceptual decision-making network. We conducted an additional ALE meta-analysis focusing on reward-related decision-making tasks. The Reward > Control contrast showed several regions including right SMG, right IFG pars orbitalis, left insula, and anterior cingulate gyrus (ACC). Several subcortical structures were found to be active including the striatum, the thalamus, and the SN, all areas that are deemed essential to the processing of reward-related information (Helfinstein et al., 2013). No clusters were found in the parietal cortex, which may seem surprising when taking the perceptual decision-making and task difficulty meta-analysis results into account. The main paradigm employed in the included reward-based decision-making studies was a monetary incentive delay (MID) task. In the MID task, the participant is instructed to respond as quickly as possible after receiving a cue that indicates the possible reward (Knutson et al., 2001). In such a task, one might argue that continuous stimulus information does not need to be accumulated, whereas in tasks such as the random dot motion paradigm where this is clearly the case. This fundamental difference in paradigms would explain why areas such as hIP3 are not consistently found in the current analysis on reward-based decision making. Comparing the perceptual and the reward-based decision-making networks only showed partial similarities as the conjunction analysis showed an overlap in the right anterior insula. The subtraction analysis revealed that the perceptual decision-making network recruited a number of unique areas compared to the reward-based decision-making network and vice versa.

In conclusion, these results indicate that the core network involved in perceptual decision-making has almost no overlap with the network involved in reward-based decision-making. The anterior insula was the only area that was found to be active in all three networks (including activity associated with perceptual task difficulty). This finding is in line with previous work that argues that the anterior insula is a key node involved in the integration of information from different sources and modalities (Ho et al., 2009; Sterzer and Kleinschmidt, 2010; Chang et al., 2013). The fact that the anterior insula is active during simple perceptual decision-making, dissociates between low and high task difficulty, and is involved in reward-based decision-making supports the hypothesis of the integrative nature of this area (Kurth et al., 2010; Menon and Uddin, 2010).

### Limitations of the current study

The present study entails several limitations. First, only a limited number of studies could be included in the current ALE meta-analyses on perceptual decision-making. After a rigorous literature search, only 18 out of 3230 potential articles were deemed suitable to be included. But even with the strict inclusion criteria, the suitable studies still varied on a large number of variables such as statistical thresholds, smoothing kernels, registration procedures to standard space, and MRI scanning parameters. While current coordinate-based meta-analysis methods cannot account for all these differences separately, ALE does estimate a spatial uncertainty per individual study thereby alleviating some of the between-studies variability arising from varying study specific parameters such as the number of subjects or the use of different brain templates (Eickhoff et al., 2009). The second limitation is the anatomical specificity of the results. The included studies only report the peak coordinates of what is most likely a larger activation area and are based on statistical procedures that include smoothing kernels ranging from 4 to 8 mm FWHM. This inherently limits the anatomical specificity of the results of both the included studies as well as the current meta-analysis. A third limitation is the lack of methodological or procedural information in some of the original studies, which limits the assessment of their similarity to other data included in the meta-analysis (Poldrack et al., 2008). For instance information regarding the use of a linear or non-linear registration normalization procedure, whether incorrect responses were included or whether the subjects responded with one or both hands was not always indicated. Fourth, the included data did not allow us to determine the precise role of the BG in perceptual decision making as, based solely on activation coordinates, we cannot distinguish different possible underlying processes that might have given rise to the BG activation in the original publications. This would require model-based imaging methods that use parametric analyses to directly link functional activation to cognitive mechanisms. While such approaches exist (Forstmann et al., 2010b; Nagano-Saito et al., 2012) the currently available meta-analysis techniques cannot combine results from parametric analyses with coordinates from purely contrast-based analyses. The final more general, limitation is that not all conducted studies are reported in the literature, as studies that fail to find significant results are typically not published. This phenomenon is often referred to as the “bias against null results” or “the file drawer problem” (Rosenthal, 1979). While this problem is common to all meta-analyses and cannot be resolved at present, initiatives such as the Open Science Framework (http://openscienceframework.org/) and pre-registered reports in journals (Chambers, 2013) will help to overcome this limitation in the future.

## Conclusions

The results of the current meta-analysis argue in favor of a frontal-parietal network involved in perceptual decision-making that is possibly complemented by the basal ganglia. While several cortical parts of this network, i.e., pre-SMA, anterior insula, pre-motor cortex and the PFm, are modulated by task difficulty, our conjunction analysis yielded only a small functional overlap between perceptual and reward-based decision-making that is restricted to the anterior insula. In contrast, the subtraction analysis revealed that a considerable number of areas that were uniquely involved in perceptual decision-making compared to reward-based decision-making and vice versa.

### Conflict of interest statement

The authors declare that the research was conducted in the absence of any commercial or financial relationships that could be construed as a potential conflict of interest.
