# Corrigendum: Brain networks of perceptual decision-making: an fMRI ALE meta-analysis

**DOI:** 10.3389/fnhum.2017.00139

**Published:** 2017-03-22

**Authors:** Max C. Keuken, Christa Müller-Axt, Robert Langner, Simon B. Eickhoff, Birte U. Forstmann, Jane Neumann

**Affiliations:** ^1^Faculty of Social and Behavioural Science, Cognitive Science Center Amsterdam, University of AmsterdamAmsterdam, Netherlands; ^2^Max Planck Institute for Human Cognitive and Brain SciencesLeipzig, Germany; ^3^Institute of Clinical Neuroscience and Medical Psychology, Heinrich Heine University DüsseldorfDüsseldorf, Germany; ^4^Research Centre Jülich, Institute of Neuroscience and Medicine (INM-1)Jülich, Germany; ^5^Leipzig University Medical Center, IFB Adiposity DiseasesLeipzig, Germany

**Keywords:** decision-making, meta-analysis, fronto-parietal-basal ganglia, corrigendum, GingerALE

## Introduction

Recently, Eickhoff et al. (2017) reported that the software version of GingerALE (2.3 http://brainmap.org/ale/) which was used for our meta-analysis published in Keuken et al. (2014) contained several severe implementation errors regarding the multiple-comparison correction. These errors resulted in a more liberal statistical threshold than was specified by the authors. With the newest release of GingerALE (2.3.6), these implementation errors should be remedied.

## Methods

In line with the recommendation by Eickhoff et al. (2017) and following the example by Smith and Delgado (2017) we re-analyzed the original data implementing the corrected multiple-comparison correction using GingerALE (2.3.6). All other statistical parameters were identical to those previously reported in Keuken et al. (2014). As expected, when applying a more stringent threshold, the major change in results pertains to a reduction in the number of significant clusters that survived correction for multiple comparisons. Here, we report results of our re-analysis, providing an adjusted original Table 4, indicating which clusters did not survive the correct statistical threshold (see Table 4).

**Table 4:** **The original Table 4 of Keuken et al. (2014) adjusted for the correct statistical multiple comparison implementation**.

**Contrast**	**Region**	**Volume**	**x**	**y**	**z**	**ALE(× 10^3^)**
Task > Control (minimum cluster size 304 mm^3^)	L pre-SMA		−	−	−	n.s.
	R pre-SMA		−	−	−	n.s.
	R insula; anterior part		−	−	−	n.s.
	R insula; anterior part		−	−	−	n.s.
	L insula; anterior part		−	−	−	n.s.
	R putamen		−	−	−	n.s.
	R inferior parietal lobule (PFop)		−	−	−	n.s.
	L middle frontal gyrus		−	−	−	n.s.
	R posterior cingulate gyrus		−	−	−	n.s.
	R inferior parietal lobule (hIP2)		−	−	−	n.s.
	R anterior occipital sulcus (hOC5)		−	−	−	n.s.
	L inferior frontal gyrus; *P. opercularis*		−	−	−	n.s.
Hard > Easy (minimum cluster size 320 mm^3^)	L pre-SMA	1,208	2	18	46	24.7
	R insula; anterior part	680	38	20	0	20.1
	R inferior frontal gyrus; *P. triangularis*	−	−	−	−	n.s.
	R precentral gyrus	752	42	4	32	25.4
	R angular gyrus; hIP3	32	28	−60	46	14
	R superior occipital gyrus; SPL		−	−	−	n.s.
	L inferior frontal gyrus; *P. opercularis*		−	−	−	n.s.
	L precentral gyrus		−	−	−	n.s.
	L precentral gyrus		−	−	−	n.s.
	L insula; anterior part	280	−32	22	4	17.6
	R precentral gyrus	128	32	−6	54	15.3
	L superior frontal gyrus		−	−	−	n.s.
	L superior frontal gyrus		−	−	−	n.s.
	L superior parietal lobule (SPL)		−	−	−	n.s.
	R inferior parietal lobule (hIP3)		−	−	−	n.s.
	L inferior parietal lobule (hIP3)		−	−	−	n.s.
	L middle occipital gyrus (hOC3v)		−	−	−	n.s.
	R middle occipital gyrus		−	−	−	n.s.
	L calcarine gyrus		−	−	−	n.s.
	R calcarine gyrus		−	−	−	n.s.
	R middle frontal gyrus		−	−	−	n.s.
	L superior occipital gyrus		−	−	−	n.s.
Reward anticipation > Control (minimum cluster size 288 mm^3^)	R caudate nucleus	10,048	12	10	−10	33
	L putamen		−12	8	−10	26.5
	L caudate nucleus		−6	2	0	23
	R pallidum		10	4	−2	17.7
	R rectal gyrus		22	12	−16	17.2
	R amygdala		22	2	−20	15
	L amygdala		−14	2	−16	14.3
	L thalamus	1,344	0	−18	10	16
	R substantia nigra	1,192	8	−16	−18	19.6
	L mammillary body		−2	−16	−18	14.1
	R inferior frontal gyrus; *P. orbitalis*	1,032	36	22	−22	14.4
	R inferior frontal gyrus; *P. orbitalis*		42	22	−14	13
	R superior medial gyrus	640	6	46	30	15.2
	L frontal orbital cortex	544	−38	14	−16	14.5
	L cerebellum; lobule VII crus II	304	−22	−74	−42	13.8
	L parahippocampal gyrus	72	−22	−26	−12	9.6
	L anterior cingulate gyrus	72	0	42	12	10
	R parahippocampal gyrus	40	42	−46	−2	9.5
	R superior medial frontal gyrus	16	6	52	16	9.3

*The structures that did not survive the correct statistical correction are now reported as not significant (n.s.)*.

## Results

For the contrast Task > Control condition, no clusters survived the multiple-comparison correction.

For the contrast Hard > Easy condition, the right pre-supplementary motor area (pre-SMA), right pre-central gyrus, right angular gyrus (hIP3) and the bilateral anterior insula were the only clusters that remained significant after correction. The biggest change was that there was no cluster in the inferior frontal gyrus (IFG), superior frontal gyrus, and occipital lobe.

For the contrast Reward Anticipation > Control condition there was no change in the reported clusters.

Given that there were no surviving clusters for the Task > Control contrast, the conjunction analysis as reported in the original Table 5 is non-informative.

## Discussion

It was surprising that the Task > Control analysis with 11 incorporated experiments resulted in no surviving clusters. The Hard > Easy condition with 13 incorporated experiments, and Reward anticipation > Control condition with 14 incorporated experiments replicate most, if not all, previously found clusters. This did not seem to be driven by the initially reported cluster size as the original Task > Control analysis reported similar cluster volumes as the other two analyses.

The changes in results should also be seen in light of recent recommendations of sample size in coordinate-based meta-analyses (Eickhoff et al., 2016). To have sufficient power for moderate effects it is recommended to include a larger number of experiments in a given contrast than we included.

## Conclusion

The original conclusion regarding a task-general network for perceptual decision-making is no longer warranted based on the corrected results in the Task > Control analysis. It thus remains an open question whether the lack of significant convergence is just a matter of limited power or whether there simply is no common network involved across the various included paradigms taxing perceptual decision-making.

To allow others to re-analyze our results and to incorporated additional experiments for sufficient statistical power, we have uploaded the raw input files. The data can be found on (https://app.box.com/s/v974c7fdo6r1o89vjy96tuktyw170ol3).

### Conflict of interest statement

The authors declare that the research was conducted in the absence of any commercial or financial relationships that could be construed as a potential conflict of interest.
